# Imatinib Treatment Induces CD5+ B Lymphocytes and IgM Natural Antibodies with Anti-Leukemic Reactivity in Patients with Chronic Myelogenous Leukemia

**DOI:** 10.1371/journal.pone.0018925

**Published:** 2011-04-18

**Authors:** Silvia Catellani, Ivana Pierri, Marco Gobbi, Alessandro Poggi, Maria Raffaella Zocchi

**Affiliations:** 1 Clinical Oncohaematology, Department of Internal Medicine, University of Genoa, Genoa, Italy; 2 Unit of Molecular Oncology and Angiogenesis, National Institute for Cancer Research, Genoa, Italy; 3 Division of Immunology, Transplants and Infectious Diseases, Scientific Institute San Raffaele, Milan, Italy; University of Palermo, Italy

## Abstract

Imatinib mesylate is a first line treatment of Chronic Myelogenous Leukemia and of a rare form of gastrointestinal stromal cancer, where the response to the drug is also linked to the immune system activation with production of antineoplastic cytokines. In this study, forty patients in the chronic phase of disease, treated with imatinib mesylate, were analyzed. Bone marrow aspirates were drawn at diagnosis, after 3, 6, 12, 18 months for haematological, cytofluorimetric, cytogenetic, biomolecular evaluation and cytokine measurement. Responder and non responder patients were defined according to the European LeukemiaNet recommendations. In responder patients (n = 32), the percentage of bone marrow CD20^+^CD5^+^sIgM^+^ lymphocytes, and the plasma levels of IgM, were significantly higher, at 3 months and up to 9 months, than in non responders. These IgM reacted with O-linked sugars expressed by leukemic cells and could induce tumor cell apoptosis. In responeìder patients the stromal-derived factor-1 and the B-lymphocyte-activating factor of the tumor necrosis factor family significantly raised in the bone marrow after imatinib administration, together with the bone morphogenetic proteins-2 and −7. All patients with high number of CD20^+^CD5^+^sIgM^+^ cells and high stromal-derived factor-1 and B lymphocyte activating factor levels, underwent complete cytogenetic and/or molecular remission by 12 months. We propose that CD20^+^CD5^+^sIgM^+^ lymphocytes producing anti-carbohydrate antibodies with anti-tumor activity, might contribute to the response to imatinib treatment. As in multivariate analysis bone marrow CD20^+^CD5^+^sIgM^+^ cells and stromal-derived factor-1 and B-lymphocyte-activating factor levels were significantly related to cytogenetical and molecular changes, they might contribute to the definition of the pharmacological response.

## Introduction

In the last years, tyrosine kinases have become the major targets of anti-cancer drugs. In particular, imatinib mesylate, an ATP competitive inhibitor of the constitutively activated ABL1 tyrosine kinase of the BCR-ABL oncoprotein, resulting from the t(9,22) reciprocal chromosomal translocation (Ph^+^), has become a first line treatment of Ph^+^ Chronic Myelogenous Leukemia (CML) [1], [2]. Due to its additional capacity to bind to the KIT and PDGFR-associated kinases, imatinib also provide an effective treatment of a rare form of gastrointestinal stromal cancer [3]. In the latter case, the response to the drug in vivo is thought to be also due to an effect on immunocompetent cells, able to produce cytokines with antineoplastic activity, including interferon gamma or type I interferons [3]–[5]. In addition, imatinib has been shown to modulate cytokine expression in human cancer-associated stromal fibroblasts, enhancing mainly interleukin (IL)-6 and the chemokine IL-8 [6]. In turn, IL-6 is known to increase the number of bone marrow (BM) immunoglobulin (Ig)M-secreting B cell, still not terminally differentiated [7]. Also, it has been reported that BCR-ABL translocation, beside determining the constitutive activation of the ABL1 kinase, inhibits the interaction between the stromal-derived factor (SDF)-1 and its receptor [8], [9]. On the other hand, defective or altered production of SDF-1, have been related to impaired B cell maturation and differentiation [9], [10].

In some patients, resistance to imatinib can occur, or develop during treatment. This is usually due to point mutations in the BCR-ABL ATP-binding site, that impede the binding of imatinib [11]. However, other mechanisms of resistance have been reported, such as BCR-ABL gene amplification, aberrations in other oncogenetic signalling pathways, and the persistence of leukemic stem cells [12]–[14]. Extrinsic factors contributing to resistance have also been hypothesized, including multidrug resistance and microenvironmental factors [14], [15].

In this study, we show that the clinical response to imatinib relates to a significant number in the percentage of BM CD20^+^ lymphocytes, co-expressing CD5 and surface (s) IgM. This phenotype correspond to that of the so called B1 lymphocytes, producing natural antibodies [16], [17]. These antibodies are increased in the BM of patients responding to the therapy, react with O-linked carbohydrates expressed by leukemic cells and induce apoptosis. In all responder patients SDF-1 and the B lymphocyte activating factor of the tumor necrosis factor family (BAFF) transiently increased in the BM plasma after imatinib administration. The enhanced production of SDF-1 and BAFF, partially coexisting with the production of the bone morphogenetic proteins (BMP)2 and BMP7, that follows imatinib administration might contribute to the observed raise in BM CD20^+^CD5^+^sIgM^+^ lymphocytes. As all these changes were not detected in patients resistant to imatinib, they might be proposed as early markers of the pharmacological response.

## Methods

### Ethic Committee Approval

This work has been performed in the context of the approved following Ethic Statements: EUDRACT 2007-005102-42 and EUDRACT 2008-004384-19; the ethic approval for our study was obtained from the ethic committee of San Martino Hospital where the biological samples of participants were taken. According to these Ethic Statements, from all participants involved in this study a written informed consent was obtained.

### Patients

Forty CML patients, all in the chronic phase of the disease, that underwent imatinib mesylate treatment (Gleevec; formerly known as STI571, Novartis Pharma, VA, Italy; 400 mg/daily), were studied at the Clinical Hematology Division (Department of Haematology and Oncology, University of Genoa). BM aspirates were drawn at diagnosis, after 3, 6, 12, 18 months, according to conventional diagnostic and monitoring procedures, upon informed consent and IRB approval, for haematological, cytogenetic and biomolecular evaluation (Table 1). BM samples from 10 healthy bone marrow donors were used for comparison. At each time point, fractions of the samples were frozen for further experiments. Samples of peripheral blood (PB) were collected as well. Plasma from BM and PB was also recovered for evaluation of cytokines. Responder (R, n = 32) patients were defined as patients that obtained either optimal (n = 18) or suboptimal response (n = 14) at any time; non responders (NR, n = 8) as patients where imatinib treatment failed by six months, according to the European LeukemiaNet recommendations [18]. Experiments were performed on fresh specimens, data collected and stored, and interpreted retrospectively once the R vs. NR status of the patients could be assessed.

**Table 1:** Characteristics of CML patients at diagnosis in chronic phase.

AgeMedian (range)	53 (22–73)
SexMales/Females	25/15
Sokal risk scoreLowIntermediateHigh	14 (36%)16 (39%)10 (25%)
Response at 3 moCHR/<CHR/noCHR^1^	18/14/**8**
Response at 6 moCCgR or PCgR/<PCgR/no CgR^1^	28/10/**4**
Response at 12 moCCgR or PCgR/<PCgR/no CgR^1^CMoR/MMoR^1^	32/**4/4**5/14

1 CHR: complete hematologic response (normalization of peripheral blood count and no palpable spleen); CCgR: complete cytogenetic response (Ph^+^ none); PCgR: partial cytogenetic response (Ph^+^ 1–35%); no CgR: no cytogenetical response (Ph^+^>95%); <:less than; <PCgR include minor (Ph^+^ 36%–66%) and minimal (Ph^+^66–90%); CMoR: complete molecular response (transcript non-detectable); MMoR: major molecular response (<0.1%). All these definitions of the clinical response have been assigned according to the European LeukemiaNet. (Numbers in bold: non responder patients, i.e. no CHR, no CgR at 3 and 6 mo; these patients underwent imatinib 800 mg/daily after 6 mo and nilotinib or dasatinib after 12 mo).

### Immunoflurescence and cytofluorimetry

Samples from BM and PB were subjected to density gradient centrifugation and purified cells were stained with the fluorescein isothiocyanate (FITC)-conjugated anti-human sIgM or anti-human IgG antibody (Sigma Chemical Co., St. Louis, MO), followed by the allophycocyanin-conjugated (APC)-conjugated anti-CD20 monoclonal antibody (mAb), the phycoerythrin (PE)-conjugated anti-CD5 mAb, purchased from BD Pharmingen Europe (Milan, Italy). Control samples were stained with APC-, FITC- or PE-conjugated irrelevant mAbs (BD Pharmingen). In other experiments, BM plasma samples obtained from CML patients, diluted 1∶2, 1∶10, 1∶80, were incubated with autologous or allogeneic leukemic cells, isolated from BM samples drawn at the onset of the disease (at that time leukemic cells in the BM were always >90%), purified by negative depletion of monocytes and of T or B lymphocytes performed using the appropriate kit of the StemCell Technology (Vancouver, Canada) and kept frozen until use. Then FITC-anti-human sIgM or anti-human IgG antibody was then added. Some samples of CML were treated with 20 µU/mL O-glycosidase (Boehringer, Mannheim, Germany) or 10 mU/mL N-glycosidase (Roche Diagnostic GmbH, Mannheim, Germany) for 4 h at 37°C before incubation with BM plasma. After washing, samples were run on a CyAn ADP cytofluorimeter (Beckman-Coulter), gated either on lymphocytes or on leukemic cells, after exclusion of cell debris on the basis of FSC and SSC, and results expressed as percentage of positive cells or as arbitrary units of mean fluorescence intensity (MFI a.u.)

### Apoptosis assay

For apoptosis vs. cytotoxicity evaluation, CML cells obtained at diagnosis as described, were incubated with autologous or allogenic BM plasma (in some experiments plasma samples incubated 30 min at 56°C, to inactivate complement, were used) for 6 h, 12 h, 24 h or 36 h at 37°C. The percentage of apoptotic cells was analyzed by staining with FITC-annexin V (AV) and PI as described. Sample were run on a cytofluorimeter CyAn ADP (Dako, Glostrup, Denmark), equipped with an argon ion and HeNe red laser to excite FITC, PE or APC, respectively, gated on the basis of side and forward scatter and apoptotic cells were evaluated as AV^+^PI^+^ cells.

### ELISPOT for cytokine detection and ELISA for immunoglobulins

BM and PB plasma of patients undergoing imatinib therapy were analyzed for the content of cytokines able to induce B lymphocyte growth and/or differentiation, such as interleukin (IL)-4, IL-6, IL-3, IL-10, IL-21 or BAFF [19]–[23], for the levels of BMP2, BMP7 and of the chemokines MCP-1, SDF-1, IP-10 and IL-8 [23], [24], by ELISPOT Multiplex kits (Bioclarma, Turin, Italy) and referred to standard curves. In some experiments, supernatants (SN) from BM cultures obtained from 6 CML patients at diagnosis and exposed in vitro to 5 µM imatinib mesylate for 3 to 14 days, were also tested for the content of SDF-1 and BAFF. Results are expressed as pg/mL.

IgM and IgG content was measured in BM plasma by ELISA, using a commercial kit (Bethyl Laboratories, Inc., Montgomery, TX, USA) containing specific antibodies against these Ig classes and HRP-streptavidin conjugated secondary antibodies. Following development with ABTS, plates were read at OD_405_, referred to a standard curve and results expressed as mg/dL.

### mRNA isolation, reverse transcription and Q-RT-PCR for SDF-1, BAFF, BMP2 and BMP7

Total RNA was extracted with TriPure (Roche Diagnostics, Milan, Italy) from cells isolated from BM samples, before and after imatinib treatment, and reverse-transcribed with random primers. In some experiments, BM specimens of 6 CML patients at diagnosis were cultured in vitro for 3 to 14 days with 5 µM of the purified imatinib mesylate (Novartis Pharma AG, Basel, Switzerland, MTA31812); cells were recovered at day 3, 5, 7 and 14 for RNA extraction. Primers for BAFF, SDF-1, BMP2 and BMP7 amplifications and probes were purchased from Applied Biosystem (userid: Hs00198106_m1, Hs00171022_m1, Hs01055564_m1 and Hs00233477_m1, respectively). Q-RT-PCR was performed on the 7900HT FastRT-PCR system (Applied Biosystems) with the fluorescent Taqman method [25]. mRNAs were normalized to 18s as a control gene, and referred to a standard curve, i.e. serial dilutions of plasmids containing cloned sequences of Abl (Applied Biosystem). After subtracting the threshold cycle (C_T_) value for 18s (Applied Biosystem) from the C_T_ values of the target genes, the ΔC_T_ values were converted with the formula 2^-ΔΔCT^ to show the fold relative increase in mRNA expression compared to levels prior to imatinib treatment, which were given the arbitrary value of 1 [26].

### Statistical analysis

Data are presented as mean±SD. Statistical analysis was performed using ANOVA for repeated measures. Pearson coefficient (r) at 99% confidence interval was calculated to correlate the number of CD20^+^CD5^+^sIgM^+^cells with the plasma concentrations of IgM or SDF-1/BAFF and the levels of transcription of SDF-1/BAFF with BMP2/BMP7. Correlation between each of these parameters and clinical response (cytogenetical response, CgR, or molecular response, MoR) was evaluated with the Spearman coefficient (r_s_) at 95% confidence interval and by multivariate regression analysis.

## Results

### CML patients responding to imatinib show high numbers of BM CD20^+^CD5^+^sIgM^+^ lymphocytes and high concentrations of BM IgM

Forty CML patients, that underwent imatinib treatment (400 mg/daily), were studied (Table 1). Among them, 32 were defined responders (R) as they obtained either optimal response (n = 18), or suboptimal (n = 14) response; eight patients were non responders (NR) as they underwent failure of treatment, or loss of response at 6 months (n = 4), according to the LeukemiaNet recommendations [18]. Most R patients (n = 19) had complete (CMoR, n = 5) or major molecular response (MMoR, n = 14) by 12 months (Table 1) and maintained it at 18 months. None of NR patients showed any of the known mutations responsible for imatinib resistance (not shown).

**Figure 1:**
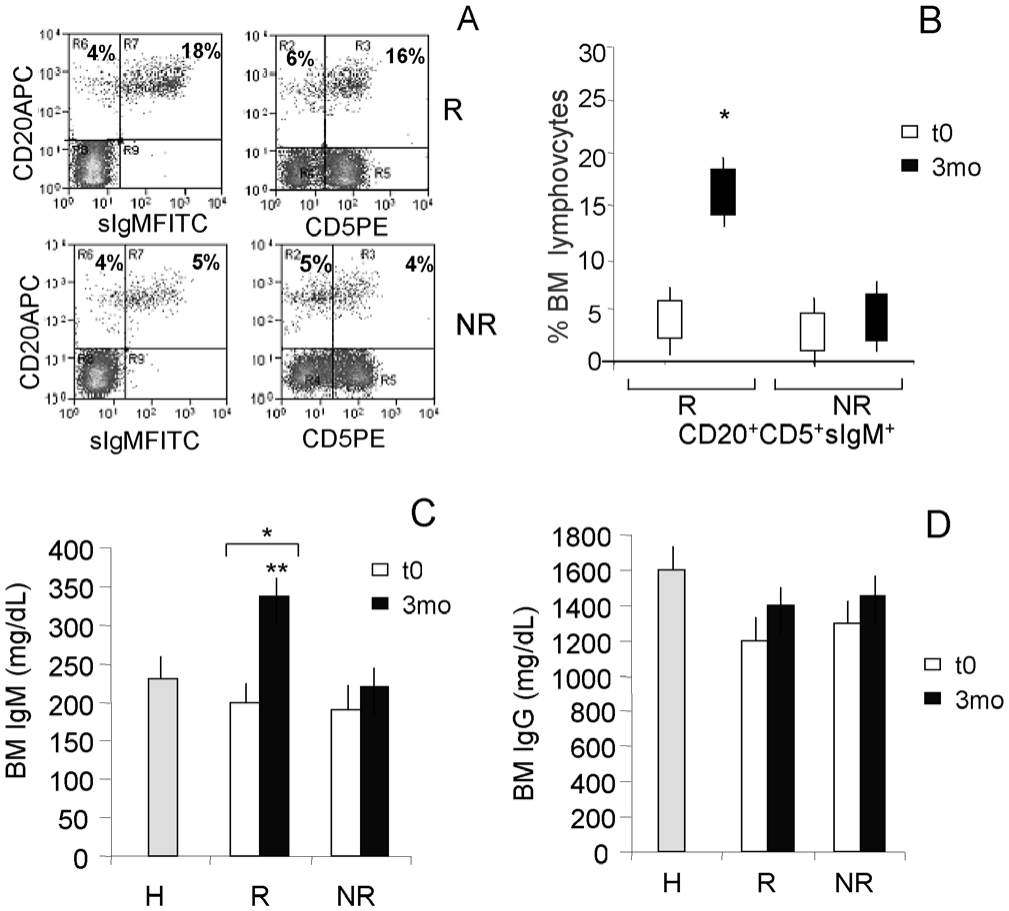
Increased CD20^+^CD5^+^sIgM^+^ lymphocytes and IgM plasma concentrations in the BM of CML patients upon imatinib therapy. BM samples were drawn after 3 mo of therapy (A–D) or at the time of diagnosis (t0) (B–D). Panels A and B: cells isolated from BM were stained with the APC-conjugated anti-CD20, followed by the FITC-conjugated anti-IgM mAb, and/or the PE-conjugated anti-CD5 mAb. Samples were run on a Cyan ADP cytofluorimeter, gated on lymphocytes and to exclude non viable cells and debris, and results expressed as log far-red fluorescence intensity (a.u.) (A) vs. log green fluorescence intensity (left dot plots) or log red fluorescence intensity (right dot plots), or percentage of positive cells (B). Panel A: R (upper dot plots) and NR (lower dot plots) patient specimens at mo 3; numbers in upper left (UL) and upper right (UR) quadrants indicate CD20^+^sIgM^−^ (UL) or CD20^+^sIgM^+^ (UR cells (left dot plots) and CD20^+^CD5^−^ (UL) or CD20^+^CD5^+^ (UR) cells (right dot plots). Panel B: BM CD20^+^CD5^+^sIgM^+^ cells at t0 and mo 3 in R and NR patients. Mean±SD from 32 R and 8 NR patients. * p<0.001 vs NR and t0. Panels C and D: IgM (C) and IgG (D) content was measured in BM plasma samples from R or NR patients or 10 healthy (H) donors by ELISA using a commercial kit containing specific antibodies against these Ig classes and HRP-streptavidin conjugated secondary antibodies. Following development with ABTS, plates were read at OD_405_, referred to a standard curve and results expressed as mg/dL. Mean±SD from 32 R and 8 NR patients. * p<0.001 vs t0; ** p<0.001 vs NR.

BM aspirates were drawn at diagnosis (t0), after 3, 6, 12 and 18 months of imatinib therapy. Cytofluorimetric analysis, performed at the mentioned time points, showed in R patients an increase of CD20^+^ lymphocytes (range 20–25% of BM lymphocytes vs 8–10% in the BM of NR patients) mostly coexpressing surface IgM and CD5 (Figure 1A). At diagnosis the number of CD20^+^ cells in all CML patients (R and NR) was lower than in healthy donors (range 8–12% of BM lymphocytes vs 18–20% in the BM of healthy donors, not shown), in agreement with other reports [27]. Besides the recovery of BM B cells, in R patients these CD20^+^CD5^+^sIgM^+^ cells represented about the 15–18% of the BM lymphocytes after 3 mo of imatinib therapy, whereas it was 5% or less in NR patients (Figure 1A and B). Also, this CD20^+^CD5^+^sIgM^+^ cell population increased, although to a lesser extent, in the PB of R patients (Figure S1 A).

BM plasma samples were also evaluated for IgM and IgG content. As shown in figure 1C, the amount of IgM significantly increased in the BM of R patients (>350 mg/dL at 3 mo vs <200 mg/dL at t0; p<0.001), compared to NR (>350 mg/dL at 3 mo in R vs <250 mg/dL at 3 mo in NR, p<0.001) and to the levels of IgM in the BM plasma from H donors (<250 mg/dL); no increase of IgG was observed (Figure 1D). The increase in IgM levels was also detectable and significant (p<0.01), although less evident, in PB samples (Figure S1 B). A significant correlation between the number of CD5^+^IgM^+^ B cells in the BM at mo 3 and the levels of IgM (r = 0.812) content in BM plasma at the same time point was found.

### BM IgM from responder CML patients react with leukemic cells

**Figure 2:**
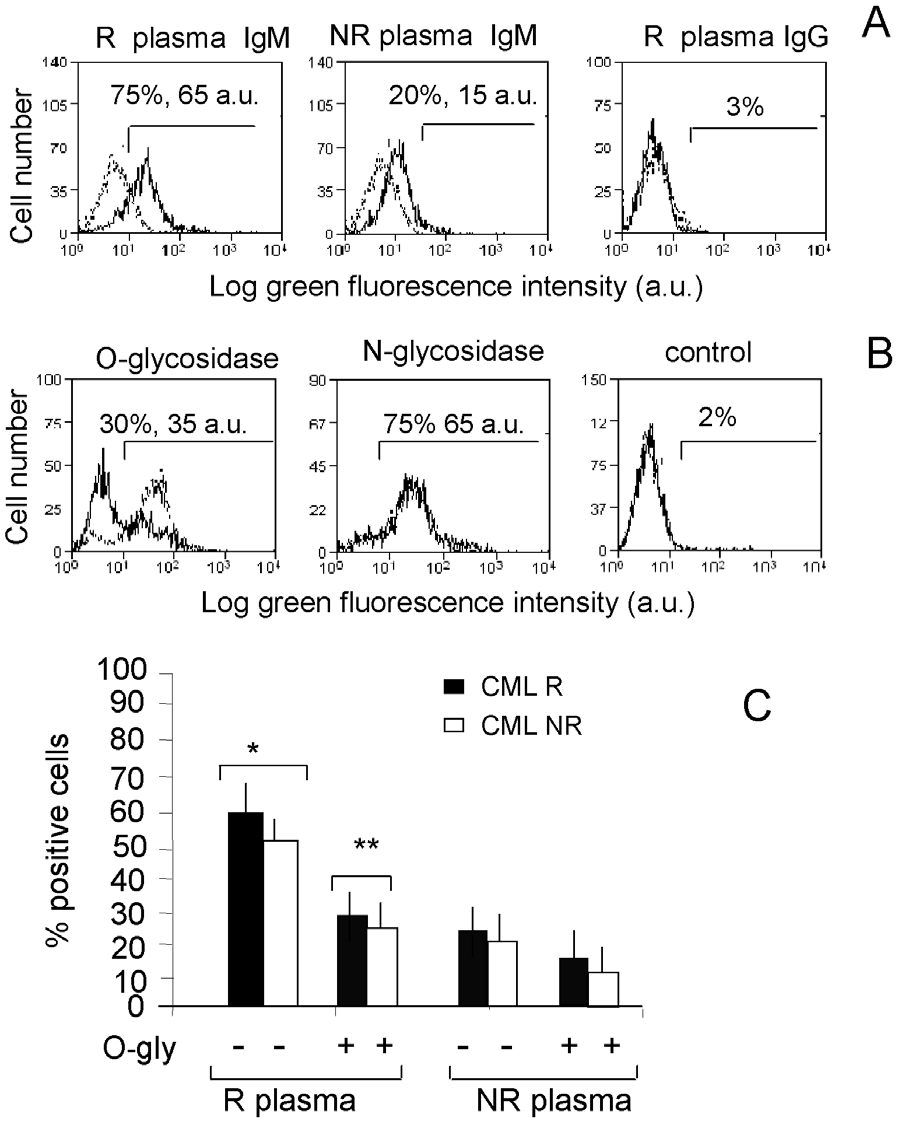
BM IgM from responder CML patients react with leukemic cells. Leukemic cells (CML) from R (panels A-C) or NR (panel C) patients were used. After incubation with BM plasma and staining with FITC anti-human IgM or IgG, samples were run on a CyAn ADP cytofluorimeter, gated on leukemic cells, after exclusion of cell debris on the basis of FSC and SSC, and results expressed as log green fluorescence intensity (a.u.) vs number of cells (A and B) or as percentage of positive cells (C). Panel A: Black lines: BM plasma samples of representative R (left and right histograms) or NR (central histograms) patients, diluted 1∶10, were incubated with R CML, followed by FITC-anti-human IgM (left and central histograms) or anti-human IgG (right histogram) antisera. Numbers in each histogram: percentage and mean fluorescence intensity (arbitrary units, a.u.) of positive cells. Grey-dotted lines: CML incubated with FITC-anti-human IgM or anti-human IgG antisera alone. Panel B: CML cells from a representative R patient were untreated (grey-dotted lines) or treated (black lines) with 20 µU/mL O-glycosidase (left histogram) or 10 mU/mL N-glycosidase (central histogram) for 4 h at 37°C before incubation with autologous BM plasma followed by FITC-anti-human IgM antiserum; right histogram: CML incubated with FITC-anti-human IgM alone. Numbers in each histogram: percentage and mean fluorescence intensity (a.u.) of positive cells. Panel C: CML cells from R (black columns) or NR (white columns) patients, untreated (−) or treated (+) with O-glycosidase were incubated with BM plasma samples from R or NR patients, followed by FITC-anti-human IgM, run on a CyAn ADP as above, and results expressed as percentage of positive cells. Mean±SD from 22 R and 8 NR patients. * p<0.001 vs NR; **p<0.001 vs (−).

Since it has been described that a population of CD5^+^ B cells can produce “natural” antibodies, mainly of IgM isotype, potentially reactive with solid tumor cells [28], [29], we investigated whether the IgM fraction of BM plasma contain antibodies reacting with leukemic cells in CML. To this aim, purified CML cells were incubated with BM plasma from R or NR patients, followed by FITC-conjugated anti-IgM or anti-IgG antiserum. Figure 2A shows that IgM reactive with autologous CML were present at 1∶10 dilution in R patients (A, left histogram) and with a very low reactivity in NR patients (A, central histogram), whereas no anti-CML IgG antibodies were found (A, right histogram). Although not shown, no reactivity was observed with mononuclear cells from healthy donors; moreover, R patients' plasma samples were still reactive at 1∶80 dilution, at variance with samples from NR patients (not shown). It has been reported that the largest proportion of IgM natural antibodies with anti-cancer reactivity is directed against tumour associated carbohydrate epitopes [29], [30]; to address this point, CML cells were pre-treated with either O-glycosidase or N-glycosidase before incubation with patients' plasma samples and staining with FITC-conjugated anti-IgM antibody. Of note, we found that pre-treatment of autologous (Figure 2B) or allogenic (Figure 2C) CML cells with O-glycosidase, at variance with N-glycosidase, strongly reduced the reactivity of patients' sera. This was mostly evident in R patients (Figure 2C), probably due to the higher amounts of IgM antibodies in their plasma.

IgM natural antibodies directed against tumor-associated carbohydrates have been shown to exert both complement-dependent cytotoxicity and apoptosis [29]–[31]. Interestingly, BM plasma (1∶10 diluted) from R patients could induce apoptosis of CML cells from either R or NR patients (about 25–30%) in 24–36 h, whereas plasma samples from NR patients or healthy (H) donors were significantly less effective (<15–20% of apoptosis by 36 h) (Fig. S1, A and B). Superimposable results were obtained with plasma samples heated at 56°C (not shown), thus making unlikely a potential complement-depending mechanism.

### Increased SDF-1/BAFF production in the BM of CML patients responding to imatinib therapy

**Figure 3:**
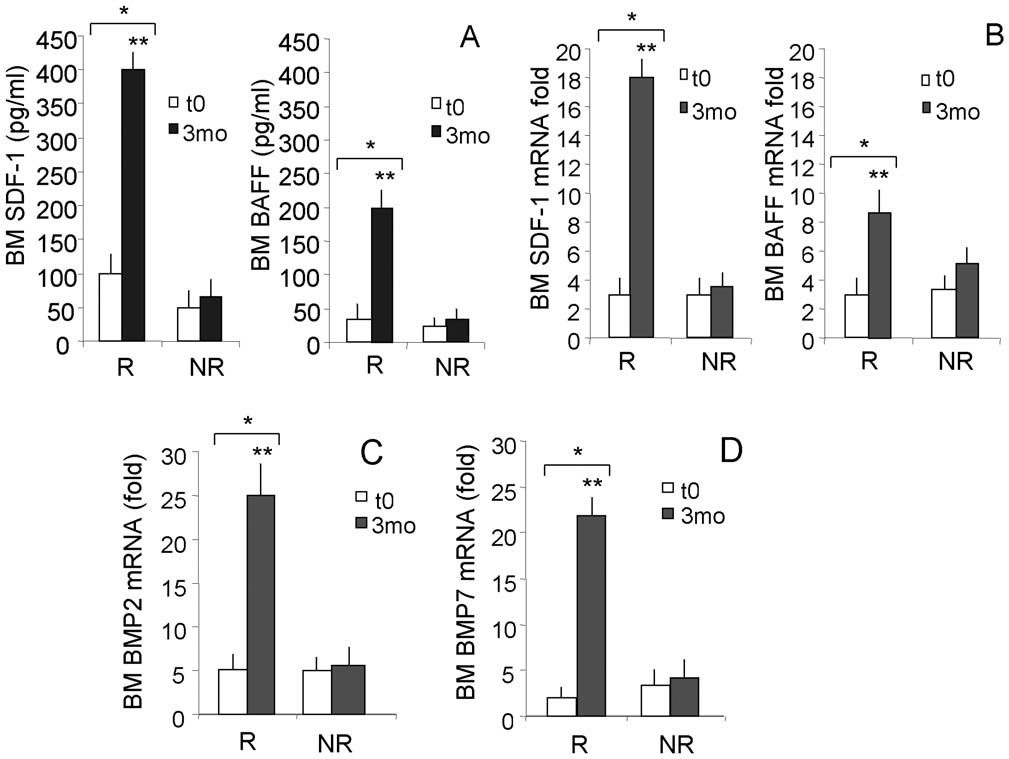
SDF-1, BAFF, BMP2 and BMP7 production in the BM of CML patients following imatinib therapy. BM samples were drawn at diagnosis (t0) or 3 months (3mo) after therapy with imatinib. Panel A: SDF-1 (left histograms) or BAFF (right histograms) content was measured in BM plasma by ELISA, referred to a standard curve and results expressed as pg/mL. Mean±SD from 32 R and 8 NR patients. * p<0.001 vs t0; **p<0.001 vs NR. Panels B–D: total RNA was extracted from cells isolated from BM samples before (t0) and after (3 mo) imatinib treatment and reverse-transcribed with random primers. Q-RT-PCR was performed with primers and probes for SDF-1 (B, left histograms), BAFF (B, right histograms), BMP2 (C) and BMP7 (D) on the 7900HT FastRT-PCR system with the fluorescent Taqman method. mRNAs were normalized to 18 s as a control gene, and referred to a standard curve. Results are expressed as the fold relative increase in mRNA expression compared to the control gene. Mean±SD from 32 R and 8 NR patients. * p<0.001 vs t0; **p<0.001 vs NR.

BM and PB plasma were analyzed, at diagnosis and at 3 mo after imatinib therapy, for the content of cytokines able to induce B lymphocyte proliferation and/or differentiation, such as interleukin (IL)-4, IL-6, IL-3, IL-10, IL-21 or BAFF and for the levels of chemokines potentially active on B cells such as MCP-1, IP-10 and IL-8 [7], [19]–[24]. SDF-1 was tested as an additional factor acting on B cell growth and differentiation [9], [10]. Of the different cyto-chemokines analyzed, SDF-1 was found to be significantly increased in the plasma of BM (Figure 3A, left panel, >400 pg/mL at 3 mo vs about 100 pg/mL at t0, p<0.001) and in part also in the PB (Figure S1 C, >150 pg/mL vs about 25 pg/mL, p<0.001) 3 mo after the beginning of imatinib therapy. More importantly, this increment was detectable in R patients, but not in NR (Figure 3A, p<0.001, and Figure S1 C). A less strong, but significant increase of BAFF was also detectable in the BM (Figure 3A, right panel, >200 pg/mL vs. 40 pg/mL, p<0.01) and PB plasma (Fig. S1 D, >100 pg/mL vs about 20 pg/mL, p<0.01) of R patients, at variance with NR patients. Likewise, increased transcription of either SDF-1 (Figure 3B, left panel) or BAFF (Figure 3B, right panel) was observed in R, at variance with NR, patients at month 3. No detectable levels of (IL)-4, IL-6, IL-3, IL-10, IL-21 or IL-8, were found (not shown). Interestingly, statistical analysis showed a significant correlation between the number of CD20^+^CD5^+^ B cells in the BM at mo 3 and the levels of SDF-1 (r = 0.761 for mRNA and r = 0.798 for protein content in BM plasma) or BAFF (r = 0.721 for mRNA and r = 0.782 for protein content in BM plasma). Of note, multivariate analysis revealed that BM CD20^+^CD5^+^ B cells at 3 and 6 mo are linked to the levels of SDF-1 (p<0.0001) and BAFF (p<0.001).

It has been reported that SDF-1 production can be stimulated by BMP2 and BMP7 [24]; thus transcripts for these proteins were analyzed in BM samples of CML patients at diagnosis and 3 mo after imatinib therapy. As shown in figure 3, transcription of BMP2 (C) and BMP7 (D) increased significantly only in R patients. Interestingly, a significant correlation between SDF-1 and BMP2 (r = 0.852) or BMP7 (r = 0.810) transcription and between BAFF and BMP2 (r = 0.796) or BMP7 (r = 0.679) transcription was found. Again multivariate analysis showed that SDF-1 and BAFF transcription are related to BMP2 (p<0.0001 and p<0.001 respectively) and BMP7 mRNA levels (p<0.0001 and p<0.001 respectively).

### Increased production of SDF-1 and BAFF by CML BM cells exposed to imatinib *in vitro*

**Figure 4:**
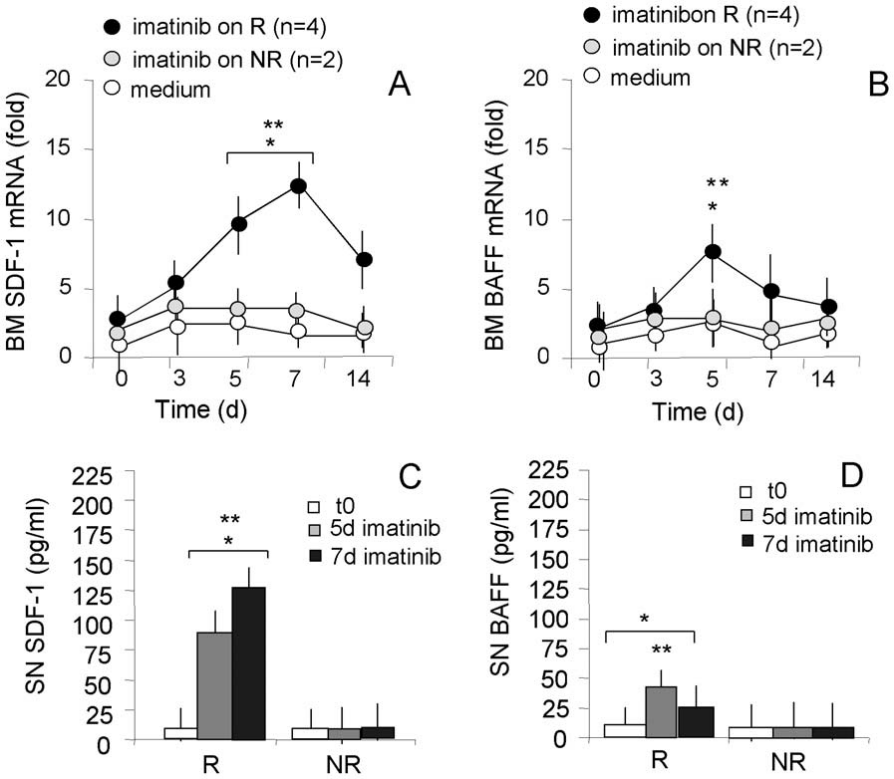
SDF-1 and BAFF production in the BM of CML patients following *in vitro* exposure to imatinib. Cells isolated from BM samples of 6 CML patients (4R and 2NR) at diagnosis (t0) were cultured in vitro for 3 to 14 days without (medium) or with 5 µM purified imatinib mesylate; cells were recovered at day 3, 5, 7 and 14. Total RNA was extracted and reverse-transcribed. Q-RT-PCR was performed with primers and probes for SDF-1 (panel A) or BAFF (panel B) on the 7900HT FastRT-PCR system with the fluorescent Taqman method. mRNAs were normalized to 18 s as a control gene, and referred to a standard curve. Results are expressed as the fold relative increase in mRNA expression compared to levels prior to imatinib *in vitro* treatment. Mean±SD from 4 R and 2 NR patients. * p<0.001 vs 3d; **p<0.001 vs NR. Panels C and D: SDF-1 (C) and BAFF (D) content was measured in SN of BM cells of CML patients (R, n = 4; NR, n = 2), at t0 or cultured as above and recovered on day 5 and 7, by ELISA kit, referred to standard curve and results expressed as pg/mL. Mean±SD from 4 R and 2 NR patients. * p<0.001 vs 3d; **p<0.001 vs NR.

To further analyze the observed effects of imatinib, BM cells obtained from 6 CML patients (n = 4 R, n = 2 NR) at diagnosis (t0) were cultured in the absence or presence of the imatinib mesylate (5 µM). Cells and SN were then recovered at different time points for the quantification of SDF-1 or BAFF mRNA by Q-RT-PCR and protein amount by ELISA. SDF-1 transcription was detectable from day 3, with a peak on day 7, and a decrease on day 14 of exposure to imatinib in cell cultures from R CML patients (Figure 4A); in turn, BAFF mRNA was transiently detectable on day 5 (Figure 4B). Significant amounts of SDF-1 protein were evidenced in the SN from R patient cultures (Figure 4C, n = 4) on day 5 (100 pg/mL vs <10 pg/mL at t0) and 7 (125 pg/mL vs <10 pg/mL at t0), while BAFF was present in the same SN in lower amounts on day 5 (Figure 4D, 50 pg/mL vs <5 pg/mL). No increase in SDF-1 or BAFF transcripts and proteins were detected in BM cell cultures from the two NR patients (Figure 4C and D). Moreover, no variations in BMP2 or BMP7 transcripts were observed in BM cells upon *in vitro* exposure to imatinib (not shown), suggesting that the whole BM microenvironment is required for imatinib to exert its complete effect.

**Figure 5:**
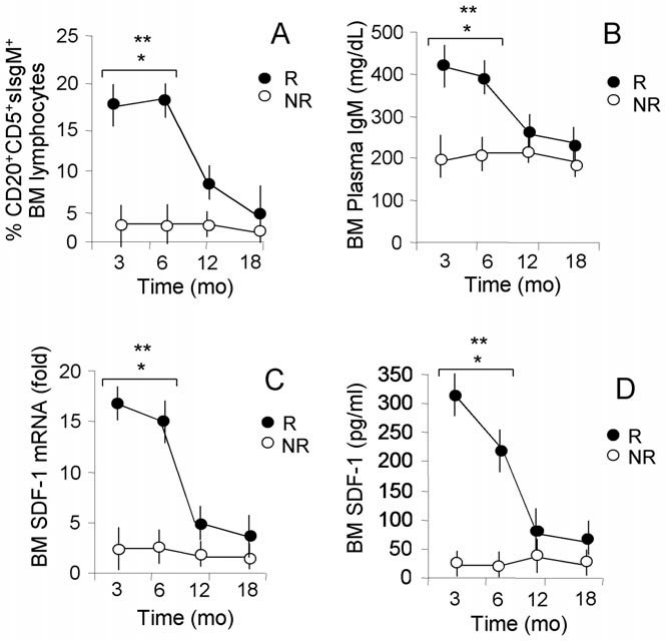
CD20^+^CD5^+^sIgM^+^ lymphocytes, IgM plasma levels and SDF-1 production in the BM of CML patients during imatinib therapy. BM samples were drawn at diagnosis (t0) or at 3, 6, 12, 18 months of therapy with imatinib. Panel A: cells isolated from BM were stained with the APC-conjugated anti-CD20, followed by FITC-conjugated anti-sIgM and by the PE-conjugated anti-CD5 mAb. Samples were run on a CyAn ADP cytofluorimeter, gated to exclude non viable cells and debris, and results expressed as percentage of positive cells. Mean±SD from 32 R and 8 NR patients. * p<0.001 vs t0; **p<0.001 vs NR. Panel B: IgM content was measured in BM plasma samples by ELISA using a specific antibody against this Ig class and HRP-streptavidin conjugated secondary antibody. Following development with ABTS, plated were read at OD_405_, referred to a standard curve and results expressed as mg/dL. Mean±SD from 32 R and 8 NR patients. * p<0.001 vs t0; **p<0.001 vs NR. Panel C: Total RNA was extracted from cells isolated from BM samples and reverse-transcribed with random primers. Q-RT-PCR was performed with primers and probes for SDF-1 on the 7900HT FastRT-PCR system. mRNAs were normalized to 18 s as a control gene, and referred to a standard curve. Results are expressed as the fold relative increase in mRNA expression. Mean±SD from 32 R and 8 NR patients. * p<0.001 vs t0; **p<0.001 vs NR. Panel D: SDF-1 content was measured in BM plasma by ELISA Multiplex kits, referred to a standard curve and results expressed as pg/mL. Mean±SD from 32 R and 8 NR patients. * p<0.001 vs t0; **p<0.001 vs NR.

**BM CD20^+^CD5^+^sIgM^+^ lymphocytes, plasma levels of IgM and SDF-1/BAFF production upon imatinib therapy: an 18months follow up.** BM aspirates were drawn at diagnosis and after 3 mo; then, follow up was carried out with BM aspirates at 6 mo and every 6 months: figure 5 depicts the results at 3, 6, 12 and 18 months. The significant high number of BM CD20^+^CD5^+^sIgM^+^ lymphocytes in R patients, early after imatinib treatment (3 mo), was observed also after 6 months (18% in R patients vs 4% in NR patients, p<0.001) and still detectable (6–8%), although decreased, after one year (Figure 5A). Also the amount of IgM remained significantly increased in the BM of R patients, up to 6 months after the beginning of imatinib therapy (Figure 5B, >400 pg/mL in R patients vs <200 pg/mL in NR patients). Along this line, high levels of SDF-1 transcription (Figure 5C) and secretion (Figure 5D), and to a lesser extent of BAFF (not shown), were detectable in the BM of R patients up to 6 months. Conversely, in NR patients, no increase of each parameter was observed during therapy (Figure 5). Statistical analysis revealed a significant correlation between clinical response to imatinib therapy (CgR at 6 mo and MoR at 12 mo), number of BM CD20^+^CD5^+^sIgM^+^ lymphocytes (r_s_ = 0.680 vs CgR, r_s_ = 0.0571 vs MoR), BM IgM plasma concentration (r_s_ = 0.640 vs CgR, r_s_ = 0.478 vs MoR) and SDF-1 levels (r_s_ = 0.658 vs CgR, r_s_ = 0.547 vs MoR) in BM plasma. All R patients showing CCgR and CMoR/MMoR at 12 months (Table 1), maintained this cytogenetic/molecular pattern and clinical remission at 18 months (not shown). Of note, multivariate analysis revealed that BM CD20^+^CD5^+^sIgM^+^ lymphocytes, BAFF/SDF1 and IgM BM plasma concentrations at 3 mo might be considered as predictive of CCR (p<0.0001) or MMoR (p<0.01) at 12 mo.

## Discussion

Imatinib mesylate is approved to treat newly diagnosed Ph^+^ CML in chronic phase and recent 6- and 7-years updates of the phase 3 International Randomized Study of Interferon and STI571 (IRIS) trial have confirmed both long-term efficacy and safety of the treatment, with an estimated overall survival rate of 86% [2], [11]. In another type of imatinib-sensitive tumor, a rare form of gastrointestinal stromal cancer, the response to the drug is also linked to the activation of the immune system with production of antineoplastic cytokines [3]–[5], thus raising the possibility that this mechanism might contribute to a good pharmacological response. Additional evidence for immune response amplification mediated by imatinib have been reported, including the enhancement of antigen presentation by dendritic cells from CML patients [32].

Herein we show that, in patients responding (R) to imatinib treatment, clinical and cytogenetic response is paralleled or preceded by early cytologic, phenotypic and molecular changes in the BM, involving the B cell compartment, that are not detectable in NR patients. A rescue of bone marrow B cell population, that is decreased in CML patients, upon imatinib therapy has been previously described [27]. We confirmed these findings and provide evidence that among B lymphocytes a subset is increased in R, but not in NR patients. In particular, a raise in the CD20^+^CD5^+^sIgM^+^ cell population is evidenced as early as 3 mo after the beginning of therapy and persists up to 6–9 mo. This population of cells is conceivably antibody-secreting, as an increase of IgM is also measured in the BM plasma of R patients. It is of note that this IgM fraction contains antibodies reactive with O-linked olygosaccharides expressed by leukemic cells and are capable to kill them by a complement-independent mechanism. Such antibodies resemble the so called “natural antibodies”, that are indeed produced by CD5^+^ B lymphocytes and in healthy donors are mainly IgM involved in complement-dependent anti-bacterial responses; natural IgM antibodies recognizing sugars, including O-linked olygosaccharides, have been reported also in patients with solid tumors, where they contribute to clear tumor cells by apoptosis [28]–[31]. A reduced concentration of gammaglobulins has been reported in the sera of a fraction of patients (one fourth) receiving imatinib treatment [33]; however, in that case, imatinib was administered at higher doses (up to 600 mg/daily) than in our study and only peripheral blood serum was considered. In the BM of CML patients, cell types other than lymphocytes or leukemic cells are present, including stromal cells.

In the BM of R patients, SDF-1 and, to a lesser extent, BAFF production is induced by imatinib both *in vitro* and *in vivo*, at variance with NR patients; both SDF-1 and BAFF have been reported to contribute to normal B cell development and maturation [9]. Thus, imatinib seems to be capable of modulating the BM microenvironment leading to conditions favourable to B cell differentiation. The reported observation that dasatinib, another tyrosine kinase inhibitor, does not restore the total B cell population in the BM of treated patients [27], might be due to the different effect of the two drugs, imatinib and dasatinib, mainly on stromal cell compartment. Indeed, dasatinib inhibits a broader range of kinases compared to imatinib [27]. It is tempting to speculate that imatinib treatment is able to “reset” the BM microenvironment, involving stromal cells possibly responsible for SDF-1 and BAFF production, that, in turn, would enhance the B cell compartment. This might be due to the therapeutic effects exerted by imatinib on the myeloid malignant progenitors [34], [35], that would be substituted by normal progenitors, capable of repopulating the BM with normal stromal as well as normal myeloid cells. In our in vitro experiments, a number of cells expressing the CD105 stromal cell marker [36] grew in cultures of BM from R patients in the presence of imatinib, at variance with NR (not shown). This hypothesis seems to be also supported by our finding that in the BM of R patients, BMP2 and BMP7 transcription are increased. Effects of imatinib on the osteoblastogenesis and bone marrow remodelling, mediated by BMP2, have been recently reported [37]. Of note, SDF-1 synthesis has been related to both BMP2 and BMP7 [24]; moreover, BAFF synthesis and release by normal myeloid cells has been described [38]. Recently, a CML-protective effect exerted by SDF-1 agonists on leukemic cells in blastic phase, has been reported [39]. However, there is no direct evidence, so far, for a role of this cytokine on CML cells in chronic phase and their progenitors; rather, SDF-1 has been shown to block colony forming units by both healthy and CML CD34^+^ cells [40]. It remains to be defined the contribution given *in vivo* by the CD20^+^CD5^+^ IgM-secreting B cells population observed in R patients to the clearance of leukemic cells. In any case they would represent the enhancement of B cell maturation and function in a restored BM, as an early sign of the response to the treatment; indeed, both the number of these cells and the levels of IgM antibodies are statistically related to the pharmacological response.

In conclusion, the BM of R patients would benefit by the renewal of the whole microenvironment, due to the efficacy of imatinib mesylate treatment, documented by BMP, BAFF and SDF-1 production, and leading to the development of the observed B cell population producing tumor-reactive IgM. These changes are transiently detectable for a period of up to 6–9 months, by which time the resetting process would be concluded, as proved by the stability of the clinical, cytogenetic and molecular response. A statistically significant correlation to the clinical, cytogenetical and molecular response in R patients was found; provided these data will be confirmed in a larger cohort of patients, we propose that they may contribute to the early identification of NR patients, and help in the choice of alternative therapeutic strategies.

## Supporting Information

Figure S1 **CD20^+^CD5^+^sIgM^+^ lymphocytes and IgM, SDF-1, BAFF content in the PB of CML patients upon imatinib therapy.** PB samples were drawn at diagnosis (t0) or 3 months after imatinib. Panel A: cells isolated from PB were stained with the APC-conjugated anti-CD20, followed by FITC-conjugated anti-sIgM and by the PE-conjugated anti-CD5 mAb. Samples were run on a CyAn ADP cytofluorimeter, gated on lymphocytes and to exclude non viable cells and debris, and results expressed as percentage of positive cells. Mean±SD from 32 R and 8 NR patients. * p<0.01 vs t0; **p<0.001 vs NR. Panel B: IgM content was measured in PB plasma samples from R or NR patients and healthy (H) donors by ELISA using a commercial kit containing specific antibodies against these Ig classes and HRP-streptavidin conjugated secondary antibodies. Following development with ABTS, plates were read at OD_405_, referred to a standard curve and results expressed as mg/dL. Mean±SD from 32 R and 8 NR patients. * p<0.01 vs t0; **p<0.001 vs NR. Panels C and D: SDF-1 (C) or BAFF (D) content was measured in PB plasma by ELISA, referred to a standard curve and results expressed as pg/mL. Mean±SD from 32 R and 8 NR patients. * p<0.01 vs t0; **p<0.001 vs NR.
(TIF)

Figure S2 **Apoptosis of CML cells upon incubation with BM plasma: comparison among R and NR patients.** CML cells, obtained from R (panel A) or from NR patients (panel B) as described, were incubated with BM plasma from R or NR patients or healthy donors (H) for 6 h, 12 h, 24 h or 36 h at 37°C. The percentage of apoptotic cells was analyzed by staining with FITC-annexin V and PI as described. Sample were run on a CyAn ADP, gated on the basis of side and forward scatter and results expressed as percentage apoptotic cells evaluated as AV^+^PI^+^ cells. Mean±SD from 22 R and 8 NR patients and from 8 H donors. * p<0.001 vs medium and H plasma; **p<0.001 vs NR.
(TIF)
