# Supplementary figures and images for: Imatinib Treatment Induces CD5+ B Lymphocytes and IgM Natural Antibodies with Anti-Leukemic Reactivity in Patients with Chronic Myelogenous Leukemia
Source: PLoS One. 2011 Apr 18;6(4):e18925. doi: 10.1371/journal.pone.0018925 (PMC3078937; doi:10.1371/journal.pone.0018925)

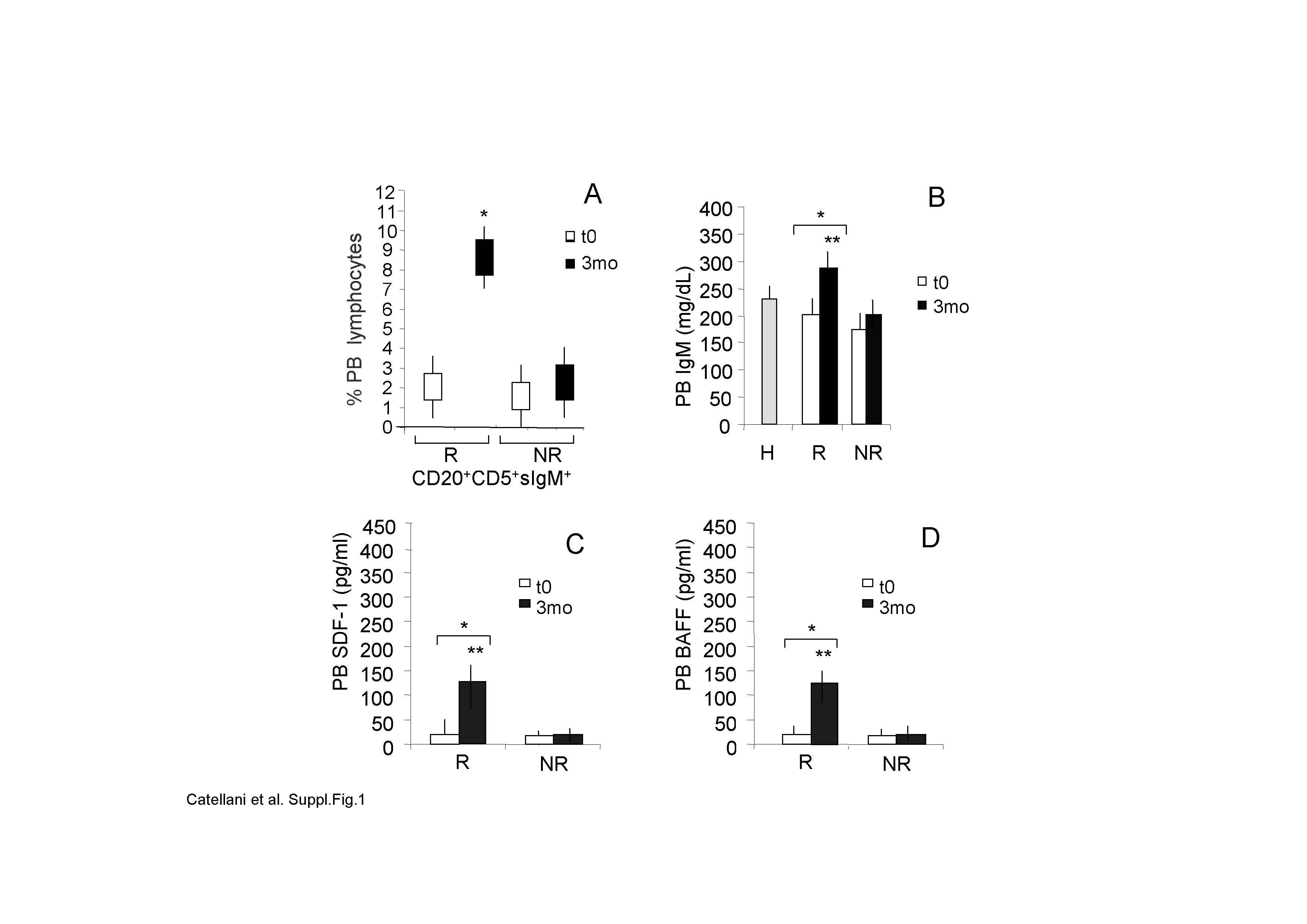

Supplement: Figure S1 — CD20+CD5+sIgM+ lymphocytes and IgM, SDF-1, BAFF content in the PB of CML patients upon imatinib therapy. PB samples were drawn at diagnosis (t0) or 3 months after imatinib. Panel A: cells isolated from PB were stained with the APC-conjugated anti-CD20, followed by FITC-conjugated anti-sIgM and by the PE-conjugated anti-CD5 mAb. Samples were run on a CyAn ADP cytofluorimeter, gated on lymphocytes and to exclude non viable cells and debris, and results expressed as percentage of positive cells. Mean±SD from 32 R and 8 NR patients. * p<0.01 vs t0; **p<0.001 vs NR. Panel B: IgM content was measured in PB plasma samples from R or NR patients and healthy (H) donors by ELISA using a commercial kit containing specific antibodies against these Ig classes and HRP-streptavidin conjugated secondary antibodies. Following development with ABTS, plates were read at OD405, referred to a standard curve and results expressed as mg/dL. Mean±SD from 32 R and 8 NR patients. * p<0.01 vs t0; **p<0.001 vs NR. Panels C and D: SDF-1 (C) or BAFF (D) content was measured in PB plasma by ELISA, referred to a standard curve and results expressed as pg/mL. Mean±SD from 32 R and 8 NR patients. * p<0.01 vs t0; **p<0.001 vs NR. (TIF) [file pone.0018925.s001.tif]

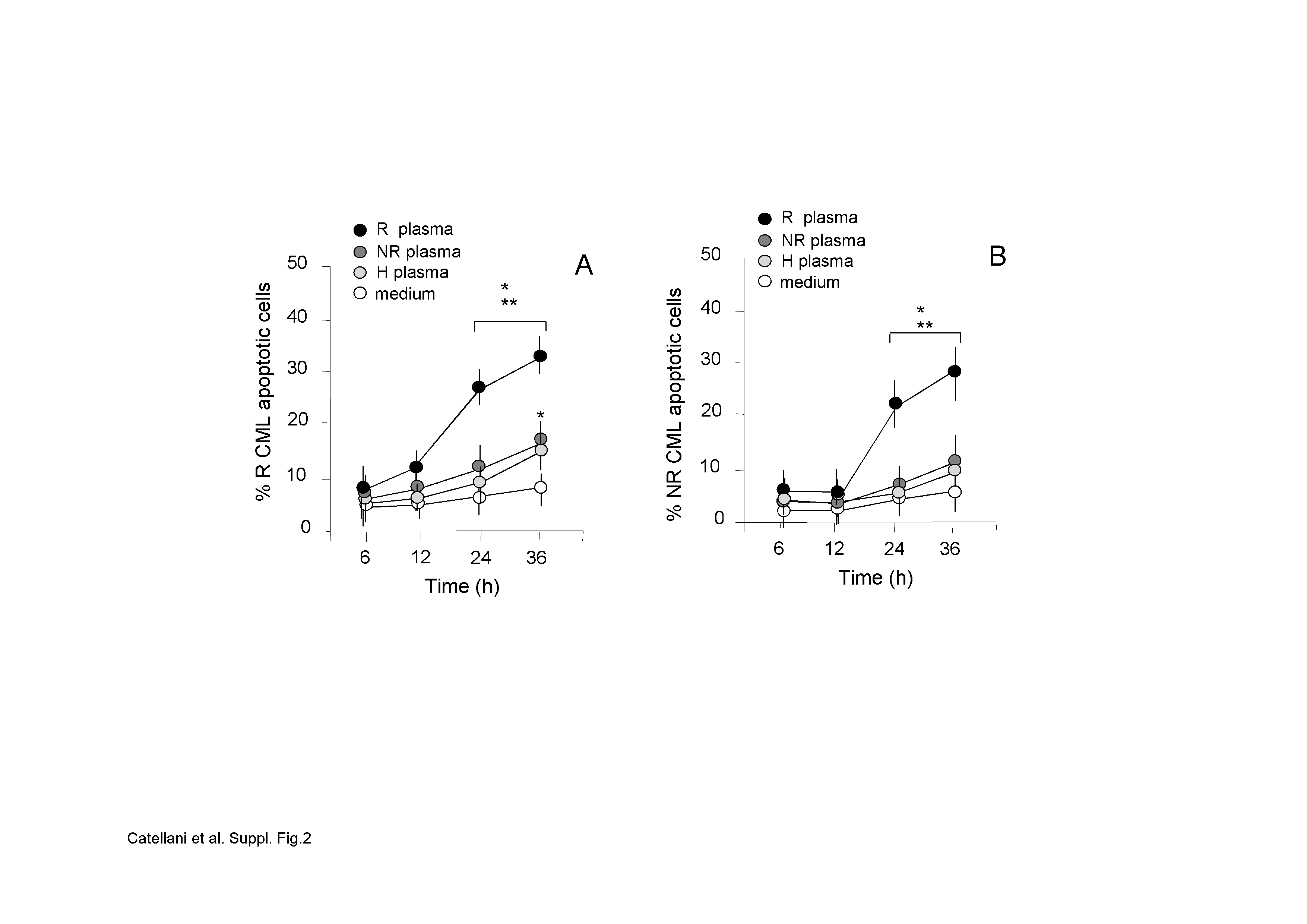

Supplement: Figure S2 — Apoptosis of CML cells upon incubation with BM plasma: comparison among R and NR patients. CML cells, obtained from R (panel A) or from NR patients (panel B) as described, were incubated with BM plasma from R or NR patients or healthy donors (H) for 6 h, 12 h, 24 h or 36 h at 37°C. The percentage of apoptotic cells was analyzed by staining with FITC-annexin V and PI as described. Sample were run on a CyAn ADP, gated on the basis of side and forward scatter and results expressed as percentage apoptotic cells evaluated as AV+PI+ cells. Mean±SD from 22 R and 8 NR patients and from 8 H donors. * p<0.001 vs medium and H plasma; **p<0.001 vs NR. (TIF) [file pone.0018925.s002.tif]
